# Targeting the Extracellular Matrix in Abdominal Aortic Aneurysms Using Molecular Imaging Insights

**DOI:** 10.3390/ijms22052685

**Published:** 2021-03-07

**Authors:** Lisa Adams, Julia Brangsch, Bernd Hamm, Marcus R. Makowski, Sarah Keller

**Affiliations:** 1 Charité—Universitaetsmedizin Berlin Corporate Member of Freie Universität Berlin Humboldt-Universitaet zu Berlin, and Berlin Institute of Health, Charitéplatz 1, 10117 Berlin, Germany; julia.brangsch@charite.de (J.B.); bernd.hamm@charite.de (B.H.); marcus.makowski@tum.de (M.R.M.); sarah.keller@charite.de (S.K.); 2 Berlin Institute of Health (BIH), 10178 Berlin, Germany; 3 Department of Diagnostic and Interventional Radiology, Technical University of Munich, Ismaninger Str. 22, 81675 Munich, Germany

**Keywords:** abdominal aortic aneurysm, extracellular matrix, pharmacological treatment, magnetic resonance imaging, molecular imaging

## Abstract

This review outlines recent preclinical and clinical advances in molecular imaging of abdominal aortic aneurysms (AAA) with a focus on molecular magnetic resonance imaging (MRI) of the extracellular matrix (ECM). In addition, developments in pharmacologic treatment of AAA targeting the ECM will be discussed and results from animal studies will be contrasted with clinical trials. Abdominal aortic aneurysm (AAA) is an often fatal disease without non-invasive pharmacologic treatment options. The ECM, with collagen type I and elastin as major components, is the key structural component of the aortic wall and is recognized as a target tissue for both initiation and the progression of AAA. Molecular imaging allows in vivo measurement and characterization of biological processes at the cellular and molecular level and sets forth to visualize molecular abnormalities at an early stage of disease, facilitating novel diagnostic and therapeutic pathways. By providing surrogate criteria for the in vivo evaluation of the effects of pharmacological therapies, molecular imaging techniques targeting the ECM can facilitate pharmacological drug development. In addition, molecular targets can also be used in theranostic approaches that have the potential for timely diagnosis and concurrent medical therapy. Recent successes in preclinical studies suggest future opportunities for clinical translation. However, further clinical studies are needed to validate the most promising molecular targets for human application.

## 1. Introduction

Abdominal aortic aneurysm (AAA) is a fatal, but often asymptomatic disease with controversial treatment and insufficient prediction of complications [1,2]. The extracellular matrix (ECM), with collagen type I and elastin as main components, is the key structural component of the aortic wall and is recognized as a target tissue for both the onset and the progression of AAA. While large and fast-growing AAAs are indicated for surgery/vascular repair, management of medium-sized AAAs remains challenging [3]. Currently, there is no effective treatment, which can slow down or prevent AAA growth.

In clinical routine, morphological criteria for the determination of the aortic diameter on ultrasound, computed tomography (CT) and MRI have been used in accordance with guidelines as evaluation criteria for therapeutic intervention or conservative therapy. By contrast, molecular imaging enables an in vivo measurement and characterization of biological processes at cellular and molecular level and sets forth to visualize molecular abnormalities at an early stage of disease, facilitating novel diagnostic and also therapeutic pathways [4]. MRI is a non-ionizing modality well suited for imaging and characterizing the relatively thin arterial vessel wall as it allows imaging with high spatial resolution and excellent soft tissue contrast [5]. By combining molecular imaging with MR target-specific probes, pathological processes can be detected and characterized in vivo [6]. This information can provide new insights into the pathogenesis of diseases in vivo and help to develop new diagnostic targets and monitor potential therapeutic success. By providing surrogate criteria for the in vivo evaluation of the effects of pharmacological therapies, molecular imaging techniques targeting the ECM may facilitate the development of pharmacological drugs. In addition, they can help to improve the clinical efficacy of medical therapies by guiding the intensity of treatment based on the properties of the molecular tissue. For example, molecular imaging of the ECM could on the one hand enable non-invasive assessment of aneurysmal tissue changes and rupture risk, and on the other hand allow to monitor the levels of key ECM proteins in response to therapeutic intervention, such as elastin and collagen [7].

In the context of AAA, development of pharmacological therapies is of high clinical relevance. It has been estimated that inhibiting the growth of an AAA by 40% could delay the occurrence of a rupture by possibly five years [8,9]. Consequently, medical treatment of AAA could act as a maintenance therapy to control AAA growth and might become a much-needed treatment option to postpone or even prevent surgical repair, especially in small to medium sized AAA. At this point, the first pharmacological approaches targeting the ECM have already been translated from preclinical studies to humans. This review addresses the latest findings in molecular imaging and therapeutic approaches related to ECM with a particular focus on studies published in recent years.

## 2. The Role of the Extracellular Matrix in AAA

In each organ, the composition of the ECM has a different three-dimensional structure and a constant pattern of remodeling to regulate tissue homeostasis [10]. Within the vascular system, the ECM is essential to resist the range of blood pressures and shear forces acting on the vessel walls. Development of AAA involves localized inflammatory response with proinflammatory cells (e.g., macrophages) and degradation/remodeling of the ECM. Disturbances in the synthesis and proteolytic degradation of the aortic structural ECM proteins, particularly collagen and elastin, have been shown to be critical to AAA pathogenesis [11]. Besides elastolysis and collagen lysis, which are reasonably well known, there is as yet little information on changes in other ECM proteins [12]. Prior proteomics analyses revealed thrombospondin 1 and 2, periostin, fibronectin und tenascin to be significantly altered proteins in the ECM of aneurysm tissue [12,13]. The protein thrombospondin 1 is a large glycosylated secretory protein with adhesive properties towards ECM components, including collagen, fibrinogen and fibronectin [13]. Periostin (fasciclin 1 family) is an ECM protein, which interacts with integrin molecules on cell surfaces, providing signals for tissue development and remodeling [14] and promoting the secretion of matrix metalloproteinases (MMPs) from cardiac cells [15]. In a mouse model of AAA, it was shown, that periostin was upregulated during the progression of AAA, particularly at times when active inflammation caused destruction of the ECM [16]. Periostin could thus function both in terms of a clinical biomarker of disease activity in AAA and in terms of a therapeutic target for patients with AAA. Fibronectin is another important glycoprotein of the aortic ECM and its expression has been reported to be increased in AAA of patients with tricuspid aortic valve stenosis or bicuspid aortic valve [17].

### 2.1. Molecular Magnetic Resonance Imaging for Identification of Potential Diagnostic Targets and Monitoring of Therapeutic Success

Molecular MRI includes both targeted probes with selective binding to molecular targets and probes that accumulate passively within cells (e.g., by phagocytosis) [18]. A major strategy of molecular MRI to achieve specificity for the target is the coupling of small molecules, peptides or antibodies with clinically approved MRI contrast agents such as gadopentetate (Gd-DTPA) or gadoteridol (Gd-HPDO3A) [19]. In addition, T2-weighted contrast agents such as iron oxide particles can be used to track or label cells.

**Figure 1:**
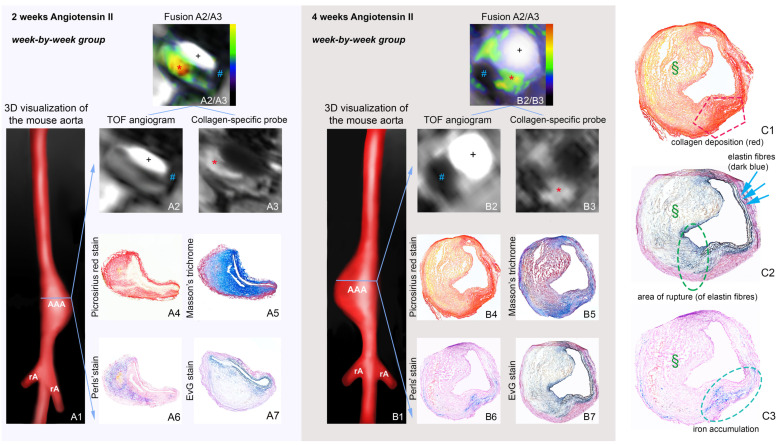
Molecular MRI (in vivo) using collagen- and inflammation-specific probes. (**A1**,**B1**) show 3D visualizations of 2- and 4-week-old AAAs. (**A2**,**B2**) show examples of oxide MRI with signal voids of different sizes for 2-week (**A2**) and 4-week (**B2**) AAAs. IR T1-weighted sequences show areas of intermediate signal enhancement of 2 weeks (**A3**) and four weeks (**B3**) AAA. (**A4**–**A7**) and (**B4**–**B7**) correspond to ex vivo histological measurements using Picrosirius red and Masson’s trichrome for visualization of collagen fibers and Perls’ staining for detection of inflammation-associated iron, confirming in vivo findings. (**C1**) illustrates compensatory collagen deposition in the aneurysmal wall after rupture. (**C2**) indicates the rupture site (green circle) with the ruptured elastin fibers and (**C3**) demonstrates iron accumulation at the rupture site. * Signal from the collagen-binding probe in the aneurysmal wall, # Signal void from the iron oxide particles, § Thrombus area. AAA suprarenal abdominal aortic aneurysm, rA renal artery, + Vascular lumen in arterial TOF. This figure was originally published in Adams et al., [21] (open access article, distributed under the terms of the Creative Commons Attribution License).

In vivo imaging of AAA wall inflammation and ECM remodeling has previously been achieved using ultrasmall superparamagnetic particles of iron oxide (USPIOs), elastin-specific MRI contrast agents (Gd-ESMA) as well as collagen- and fibrin-binding probes [20,21,22,23,24]. One advantage of USPIOs is that the substance is clinically approved and can therefore already be used in human studies. The principle of USPIO MRI is that these particles are taken up by phagocytic cells, especially macrophages, and allow visualization and quantification of inflammatory processes in the aortic wall using T2/T2* sequences. In angiotensin-II (Ang-II) infused apolipoprotein E (ApoE) -/- mice, the combined use of USPIO and EP-3533, a collagen-specific gadolinium-bound probe, allowed evaluation of ECM remodeling, inflammatory activity, and prediction of rupture events (refer to Figure 1) [21].

In a recently published prospective multicenter cohort study (MA3RS) including 342 patients with abdominal aortic aneurysms, USPIO enhancement was associated with higher risk of aortic rupture or repair, reduced event-free survival from aneurysm rupture or repair, and aneurysm expansion, although it was not independent of clinical risk factors and thus had limited additional value beyond current clinical risk prediction [25]. In a sub-cohort of the MA^3^RS study, USPIO enhancement showed no correlation with computed tomography angiogram (CTA) predictions of stress areas, so that localization of rupture-prone areas in the clinical setting has not yet been successful [26].

A further crucial process in AAA development, related in part to inflammation, is remodeling of the ECM. Dysfunctional ECM remodeling may reduce wall stability and promote AAA formation. Gd-ESMA is an elastin-specific MRI probe that showed high potential in quantitative imaging of vascular diseases [27]. By specific binding of elastin, molecular MRI allowed prediction of the site of rupture before aortic dilatation and visualization of inflammatory processes in the development of AAA in a mouse model [27,28].

**Figure 2:**
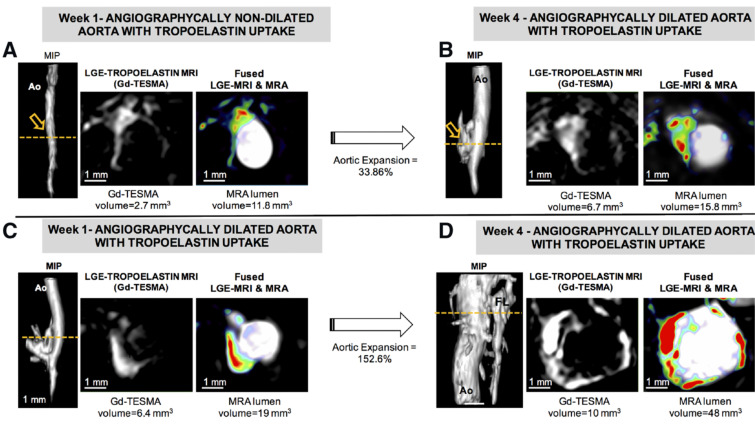
Gd-TESMA MRI shows increased tropoelastin expression in the dilated aortic segments (**C**,**D**). (**A**–**D**) MRA and late gadolinium enhancement (LGE)-MRI of a control (**A**,**B**) and an Ang II-infused ApoE-/- mouse with aortic dilatation (**C**,**D**) scanned with the tropoelastin contrast agent. Fusion of MRA and LGE-MRI images of an Ang II-infused ApoE-/- mouse after administration of Gd-TESMA show that the uptake of tropoelastin is restricted to the dilated aortic wall. Abbreviations: Ao, aorta; LRA: left renal artery; MIP, maximum intensity projection; RRA, right renal artery. Adapted from: Lavin et al., [29] (open access article, distributed under the terms of the Creative Commons Attribution License).

Another protein that may be increased in AAA due to dysfunctional ECM remodeling is tropoelastin, a monomeric precursor of cross-linked elastin [29]. A recent study using quantitative molecular tropoelastin (Gd-TESMA)- enhanced MRI found that it could identify dysfunctional ECM remodeling by being specifically expressed in regions of AAA and correlating with AAA development and expansion (Figure 2) [29].

MMPs are also associated with changes in the ECM and the development of AAA. P947 is a gadolinium (DOTA)-binding molecular probe that specifically binds to MMPs, particularly MMP-2 and MMP-9. In AAA-induced wistar rats, P947 MRI images showed good colocalization of the sample with MMPs in wall areas of inflammatory events [30].

**Figure 3:**
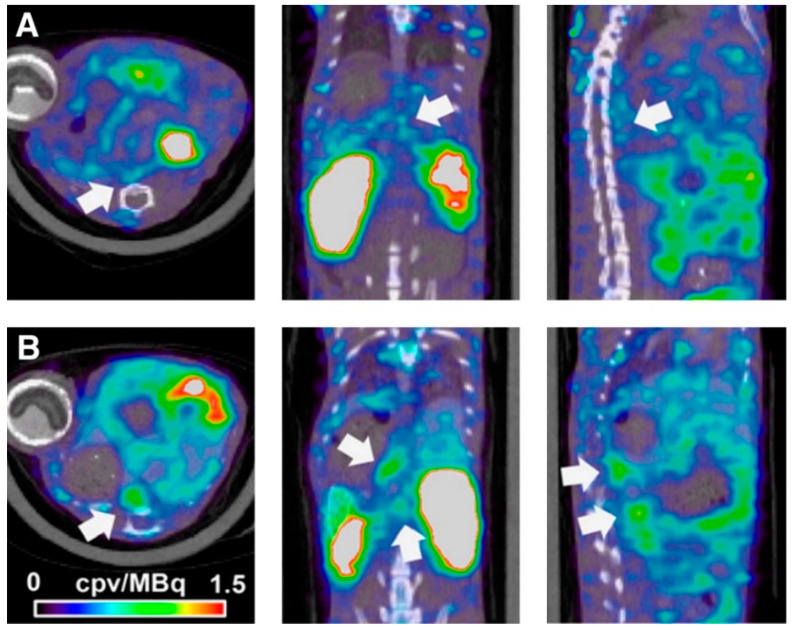
Example images for 99mTc-RYM1 imaging of AAA. A and B show examples of fused 99mTc-RYM1 SPECT/CT images from mice with little ECM remodeling (**A**) and aneurysm (**B**) groups, classified on the basis of visual in situ analysis of the abdominal aortae. Transversal (left), coronal (middle), and sagittal (right) views are provided. Arrows indicate the areas of maximal tracer uptake within the abdominal aortae. Adapted from Toczek et al. [32] © SNMMI.

MMP tracers have also been developed for nuclear imaging. The ^99^mTc-labeled homolog, RP805 predicted vessel expansion and rupture probability in Ang-II-induced murine AAA [31]. Another study investigated the ^99^mTc-labelled pan-MMP inhibitor RYM1 and demonstrated a higher uptake of the tracer in AAA compared to nondilated aorta [32]. In addition, RYM1 enabled detection of inflammation (correlation with CD68 expression) and ECM remodeling (correlation with MMP activity) (Figure 3) [32].

A recent study by *Yao* et al. investigated a smart activatable MRI nanoprobe to target MMP in early-stage AAA in a mouse model and found that their probe allowed for the detection of MMP activity within the aneurysmal wall, thus representing a potential noninvasive method to predict the risk of rupture in AAA [33].

More consistent results were achieved in preclinical studies on integrin-targeted tracers. Integrin α_v_β_3_ is upregulated in proliferating endothelial cells, VSMC and macrophages [34,35]. NC100692, a ^99^mTc-cyclic RGD tracer for microSPECT-CT displayed increased uptake in murine carotid aneurysms and a correlation with inflammatory activity [36]. English et al. recently developed a specific agent of chemokine receptor type 2 (CCR2) with the PET tracer ^64^Cu-DOTA-ECL1i [37]. CCR2 is expressed in macrophages/monocytes and mediates the migration of leukocytes to the inflammatory event in the vessel wall after injury. In induced AAA in Sprague-Dawley rats, ^64^Cu-DOTA-ECL1i showed significantly increased uptake compared with sham controls and compared with aneurysms that did not rupture during progression [37].

### 2.2. Pharmacologic Treatment Strategies Targeted to the ECM

Experimental targets for pharmaceutical AAA stabilization, that target the ECM, are thrombospondin inhibitors [38], cysteine protease inhibitors [39], serine protease inhibitors [40], protease inhibitors such as MMP inhibitors [41], and interleukins [42]. Other potential targets include inhibition of c-Jun N-terminal kinase as well as miR-29b (microRNA), both of which demonstrated a reduction of AAA via modulation of the ECM metabolism [43,44].

In humans, elevated thrombospondin-1 (TSP-1) was associated with MMP activitaion, ECM degradation as well as tissue infiltration [45]. Cysteine cathepsins (Cat) are a diverse group of proteases that are abundant in VSMCs, macrophages and endothelial cells of atherosclerotic plaques and aneurysmal lesions. Among the cysteine cathepsins, Cat S directly modulates inflammatory and immune responses and apoptosis of VSMCs, whereby elevated expression levels in the vessel wall and plasma of human AAA were recently confirmed [46]. In AAA, blockade of Cat S induced ECM degradation is still in the preclinical phase [47,48]. Recent preliminary preclinical studies in murine models showed promising results for two serine protease inhibitors, serpina3n (SA3N)—a potent inhibitor of granzyme B [40] and ulinastatin [49].

#### 2.2.1. MMPs as Pharmacologic Treatment Targets in AAA

Aneurysmal collagen and elastin degradation is caused by a number of endopeptidases called MMPs. MMPs belong to a family of enzymes whose main function is the degradation of ECM components and the disruption of tissue organization [50,51]. MMP inhibition was previously identified as a potential pharmacotherapeutic approach for limiting formation and progression of AAA [8]. MMPs degrade the many components that are actively involved in the remodeling/degradation of structural ECM components, including elastin, collagens, proteoglycans and glycoproteins [52]. In healthy tissues, MMPs are tightly regulated by specific inhibitors, also referred to as the tissue inhibitors of metalloproteases or TIMP [52,53]. Previous research indicated an imbalance between MMPs and TIMPs, resulting in an increase of proteolytic activity with subsequent degradation of ECM structural proteins and a weakening of the aneurysmal wall with higher risk of rupture [54,55]. During aneurysm formation, expression of some MMPs, such as collagenase-1 (MMP-1), gelatinase B (MMP-9) or macrophage elastase (MMP-12) is upregulated [56,57]. The development of AAAs also appears to depend on the type of MMP in the aortic tunica media and different types of MMPs are expressed in different phases of AAA development [58,59]. A recent study suggested that plasma levels of MMP-9 decrease after exclusion of AAA from circulation [60].

Being among the most abundant elastolytic proteinases produced by human tissue and aneurysm-infiltrating macrophages, MMP-9 has gained particular interest. In a molecular MRI study, Bazeli et al. reported the potential value of an MMP-targeted probe (P947) in rats. P947 was shown to co-localize with markers of inflammation and MMP activity in the area of expanding AAA [30].

MMP-12 is the most upregulated MMP in AAA and may therefore be particularly valuable for prediction of AAA progression and rupture risk. Gona et al. recently investigated the MMP-12-inhibitor ^99^mTc-AGA-2 in murine models, for which they demonstrated specific binding to MMP-12 through ex vivo competition, and found it to be significantly increased in AAA compared to healthy aortae [61].

##### Statins for Reducing MMP Levels

Katsuki et al. recently investigated the use of pitavastatin in a mouse model of AAA and found that it could inhibit AAA formation, being associated with reduced macrophage accumulation, MMP activity and elastin degradation [62]. However, there are conflicting data with regard to the effects of statins in reducing AAA growth and rupture.

##### Doxycycline as a General MMP Inhibitor

The tetracycline antibiotic doxycycline has been studied in the context of AAA for many years. In a series of earlier preclinical studies doxycycline effectively interfered with aneurysm formation or growth [63,64]. Previous research reported a reduction of MMPs in the aortic wall and a consecutive improvement of the proteolytic balance [65]. However, in contrast to the successes in preclinical studies, the Pharmaceutical Aneurysm Stabilization Trial (PHAST) trial found no effect after 18 months of doxycycline treatment (100 mg/day) [66]. This was confirmed by a recently published randomized clinical trial by Baxter et al. (Non-Invasive Treatment of Abdominal Aortic Aneurysm Clinical Trial (N-TA^3^CT)), reporting that doxycycline (200 mg/day) did not reduce AAA growth at 2 years as compared to placebo in patients with small infrarenal AAA [67]. While it was previously argued that the doxycycline dose used in the PHAST trial was too low to have an effect on AAA, the dose in the N-TA^3^CT trial corresponded to circulating levels of doxycycline that were required to reduce AAA growth in preclinical studies with mice [67]. The promising results of the preclinical studies could therefore not be confirmed in human clinical trials.

##### Other MMP-Targeting Drugs

Pentagalloyl glucose (PGG) is a polyphenolic tannin. In a mouse model of AAA, twice IV application of nanoparticle-loaded PGG (EL-PGG-NPs) showed a reduction of MMP-9, MMP-2 and of macrophages in the vascular media and restored the elastic lamina [68]. Fucoidan is a sulfated polysaccharide that can be extracted from brown seaweed. The substance is believed to have antithrombogenic, immunomodulatory, anticoagulant and antihypertensive properties. In Ang-II induced murine AAA, fucoidan significantly suppressed MMP-9 und MMP-2 activities, thereby reducing elastin degradation. In vitro, fucoidan was shown to attenuate Ang-II-induced phosphorylation of proinflammatory nuclear factor κB p65 and c-Jun N-terminal kinase activation, as well as reactive oxygen species (ROS) production [69]. Neither PGG nor fucoidan have so far been tested in humans.

#### 2.2.2. ADAMs/ADAMTS Inhibition

ADAMs and ADAMs with a thrombospondin domain (ADAM-TS) are zinc-dependent endopeptidases and belong to the proteolytic enzyme family, involved in ECM degradation and closely related to other MMPs [70]. They both have similar structural domains and play a variety of biological roles. A previous study suggested that ADAM-17 was upregulated in AAA [71]. In ApoE-deficient mice, deficiency of ADAMTS-4 resulted in reduced aortic dilatation and aortic rupture [72]. Fava et al. suggested that mice expressing truncated ADAMTS-5 (without the catalytic domain) showed an increased aortic dilatation compared to wild-type control animals [73]. To this end, Aggrecan cleavage by ADAMTS-5 has also been suggested to be important for normal aortic wall development, with increased aortic dilatation in case of altered ADAMTS-5 proteolytic profiles [74]. In human aneurysmal aortas, it could be shown that several members of the ADAMTS family, especially ADAMTS-1 are downregulated compared to control aortas, while no effect of ADAMTS-1 on aneurysm growth could be demonstrated in a transgenic mouse model [75]. The results of the aforementioned studies suggest a complex role for ADAMTS in AAA: while its role in the degradation of aggrecan and other proteoglycans is necessary for optimal physiological ECM remodeling, its loss may lead to protection or aggravation of aortic aneurysm in small animal models. ADAMTS-1 has not yet been studied in humans.

#### 2.2.3. MikroRNA Inhibition

MicroRNAs (miRNA) are small, 19–24 nucleotide, highly conserved non-coding RNAs that are involved in gene regulation by binding to the 3′ untranslated region of mRNA, thereby inhibiting translation. Through this mechanism, miRNAs are involved in diverse processes of cellular tumorigenesis, apoptosis, and differentiation [76]. In addition, miRNAs play regulatory roles in various cardiovascular diseases such as AAA [77]. In this context, numerous preclinical and clinical studies have been published with divergent expression level of the different miRNA subtypes, presumably due to methodological variances. The miR-29 family, consisting of miR29a-c, is believed to promote fibrosis through regulation of its downstream target genes [78]. Targets include collagen as well as fibrillin and elastin, all key components of the aortic wall. In addition, MMP9 and MMP2 have been identified as direct targets of miR-29b [79]. The effect of miR-29b on the development of aortic aneurysms was studied in PPE-induced AAA C57BL6 and Ang-II-induced AAA in ApoE -/- mice. In vivo administration of locked nucleic acid anti-miR-29b increased collagen expression and resulted in a significant reduction in AAA progression, whereas induced overexpression of miT-29b via a lentiviral vector led to increased AAA expansion and rupture rate, thus providing a potential target for therapeutic manipulation [80].

**Figure 4:**
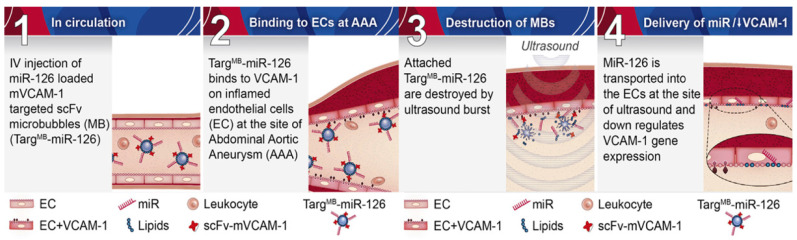
Demonstration of the theranostic TargMB-miRNA effect on VCAM-1 expression in AAA. Abbreviations: EC: endothelial cells; miRNA: microRNA; scFc-mVCAM-1: single-chain antibody targeting mouse VCAM-1; VCAM: vascular cell adhesion molecule-1. Adapted from Wang et al., [81] (open access article, distributed under the terms of the Creative Commons Attribution License).

Another potential therapeutic target is miRNA-126 with a capability to downregulate vascular cell adhesion molecule-1 (VCAM1) and thereby reduce leukocyte adhesion with a subsequent anti-inflammatory effect [81]. *Wang* et al. used ultrasound microbubbles coupled with a VCAM-1-targeted single-chain antibody (scFvmVCAM-1) and a miRNA-126mimic (M126) as carriers, enabling a theranostic approach (TargMB-M126) with simultaneous molecular imaging and targeted therapy of AAA in a mouse model of AAA [81] (please also refer to Figure 4).

Generally, miRNA appear to contribute to AAA pathophysiology, with some showing great potential for use as biomarkers or as therapeutic targets. So far, miRNAs have not yet been translated into human research. However, first results from a human relevant disease model with LDLR-/- minipigs using a custom-designed drug-eluting balloon (DEB) indicated that anti-miRNA29b treatment was a powerful therapeutic option to limit AAA progression [82]. In addition, the approach to use DEB for local delivery of a microRNA therapeutic could be a significant advantage as well as a big step towards avoiding documented side effects in non-targeted organ systems, such as kidney and liver [82].

#### 2.2.4. Interleukins

The degradation of ECM components is induced in part by cytokines secreted by inflammatory and mesenchymal cells. In this context, macrophages in particular act as important releasers of a variety of proinflammatory cytokines, including tumor necrosis factor α (TNF-α), interleukin (IL)-1β, IL-6, IL-12/IL-23 [83]. Apart from cytokines, macrophages also release MMPs and therefore contribute further to ECM degradation [84]. During AAA development, macrophages develop different phenotypes with distinct functions, consisting of classically activated macrophages with expression of pro-inflammatory cytokines and alternatively activated macrophages, which are involved in ECM remodeling and repair [83]. Previous clinical studies suggested classically activated macrophages were predominant in early stages of AAA, while alternatively activated macrophages are increased in late stage disease [85,86].

Increased production of IL-6 causes degradation of the ECM with an increased activity of MMP-9 and a consecutive aortic dilatation. IL-6 deficiency was previously demonstrated to improve AAA pathology by partially preserving ECM structure through a reduction of MMP-9 expression, whereas it could neither prevent rupture nor increase survival [42].

Inhibition of IL-12 and IL-23 has been shown to inhibit macrophage expansion and, in particular, to reduce the expression of macrophage-associated inflammatory mediators and the activity of MMPs [87]. Therefore, blockade of the IL-12/IL-23 axis has been proposed as a treatment strategy to halt the progression, especially of small aneurysms [87].

**Figure 5:**
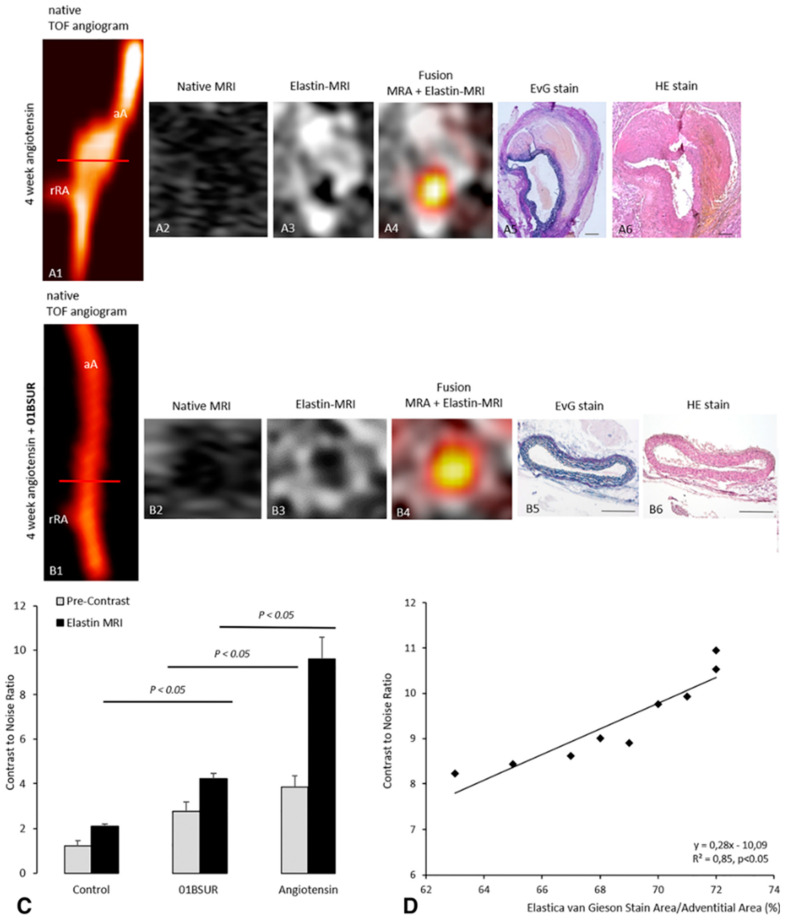
(**A1**–**A4**) and (**B1**–**B4**), in vivo molecular MRI of ECM of the aortic wall. 3D time-of-flight angiograms of the suprarenal abdominal aorta including the right renal-artery of a male ApoE-/- mouse after 4 weeks of Ang-II infusion (**A1**). After 4 weeks of Ang II infusion, an AAA with strong signs of ECM remodeling developed, which was observed in vivo by MRI after administration of the elastin-specific probe (**A3**,**A4**) and ex vivo by histological analysis (**A5**,**A6**). The abdominal aorta of a male ApoE-/- mouse treated with the anti-murine IL-1β-antibody 01BSUR shows no pathological changes of the aortic wall in vivo TOF angiogram (**B1**), native MRI (**B2**) and T1-weighted sequences using the elastin-specific probe (**B2**,**B3**) and corresponding ex vivo histology (**B5**,**B6**). (**C**), in vivo MRI signal measurements and ex vivo quantification of gadolinium-based elastin-specific probe. Contrast-to-noise ratio (CNR) values before and after the before and after the administration of the gadolinium-based elastin-specific MR probe showed a significant increase in CNR in the aortic wall in mice of the Ang-II + 01BSUR group, Ang-II group and control group. Mice in the Ang-II group showed the strongest signal enhancement, which may be explained by a strong remodeling and expression of elastic fibers in the aneurysm wall. (**D**), in vivo CNR measurements showed a strong correlation with ex vivo EvG staining on corresponding histological sections. Scale bars represent 200 µm. TOF: Time-of-flight, EvG: Elastica van Gieson staining, Elastic fibers are stained blue-black; HE: Hematoxylin-Eosin-staining; MRA: magnetic-resonance-angiography; aA: suprarenal abdominal-aorta; rRA: right renal-artery. Figure adapted from Brangsch et al. [28] (open access article, distributed under the terms of the Creative Commons Attribution License).

Interleukin-1β functions as a gatekeeper of inflammation and contributes substantially to the progressive destruction of aortic ECM proteins during the development of AAA. Disruption of the inflammatory pathway by neutralizing IL-1β represents a promising new therapeutic target for AAA therapies [88]. In a mouse model of AAA, Brangsch et al. previously investigated the effect of the IL-1β inhibitor 01BSUR in a mouse model of AAA and found that molecular MRI allowed for both early visualization and quantification of the anti-inflammatory effects of 01BSUR (Figure 5).

In addition, they suggested that molecular imaging allowed for differentiation between responders and non-responders early after initiation of therapy [89].

In humans, effect of IL-1β neutralization through monthly subcutaneous canakinumab for twelve months was evaluated (*n* = 65), but the trial was terminated afterwards for reasons of futility as no difference in aortic diameter expansion could be seen between canakinumab- and placebo-treated patients (both groups: 2.5 mm/y) [89].

## 3. Discussion and Outlook

Over the past 30 years, a variety of promising gadolinium-based molecular tracers have been developed and their utility confirmed in proof-of-concept animal studies. Nevertheless, only non-targeted contrast agents have made it to clinical approval for use in humans. A challenge in clinical translation is that toxicological testing is time-consuming and costly, requiring substantial financial resources. This contradicts the expected revenues, which are lower for molecular tracers than for clinically proven nonspecific contrast agents due to their limited application in specific settings. Nonetheless, these markers play a prominent role especially in preclinical research and, if validated, can non-invasively image new therapeutic approaches, for example targeting ECM remodeling. In this context, the elastin-specific MRI probe Gd-ESMA and tropoelastin-specific MRI probe Gd-TESMA appear promising, as they can specifically detect the effect of therapeutic strategies affecting the elastin content in the aortic wall via their different pathways. Another novel approach are multitarget probes, which could allow for concurrent imaging of different pathophysiological processes at the molecular level. Molecular agents may also be used within theranostic approaches with the potential for timely diagnosis and concurrent medical therapy. Here, the use of miRNA-based targets is a novel and promising method.

Currently, there is no established pharmacologic therapy for the treatment of AAA. The apparent discordance between successes in preclinical studies and partly disappoint results in clinical trials suggests an incomplete understanding of the various pathological processes involved in the development of AAAs and points to possible inadequacies in animal models of the disease. To increase future therapeutic strategies, molecular imaging markers that can specifically visualize and quantify targets of pharmacological agents in ECM in a non-invasive manner hold promise for a better reflection of the pathophysiological processes behind the disease and during therapy. In addition, recent preclinical approaches in large animal models represent a promising option for evaluating therapeutic strategies and create better options for multiparametric functional imaging, which seems to be limited in parts by the low volume in small animal models. With the increasing availability of image-based molecular biomarkers and promising pharmacotherapies, as well as their evaluation in large animal models of AAA and initial human clinical trials, there is reason to hope that molecular therapeutic approaches targeting ECM will enter clinical research in the coming years.

## Data Availability

Not applicable.

## Abbreviations

AAA	Abdominal aortic aneurysm
ApoE	Apolipoprotein E
CCR2	Chemokine receptor type 2
ECM	Extracellular matrix
FDG	Fluorine-18-2-deoxyglucose
GZMB	Granzyme B
IL	Interleukin
MMP	Matrix metalloproteinases
MRI	Magnetic Resonance Imaging
PET	Positron emission tomography
PGG	Pentagalloyl glucose
PPE	Porcine pancreatic elastase
ROS	Reactive oxygen species
TGF	Transforming growth factor
TIMP	Tissue inhibitors of metalloproteases
USPIO	Ultrasmall superparamagnetic particles of iron oxide
VEGF	Vascular endothelial growth factor
VSMC	Vascular smooth muscle cells
